# Efficacy and Safety of Vadadustat for Anemia in Patients With Chronic Kidney Disease: A Systematic Review and Meta-Analysis

**DOI:** 10.3389/fphar.2021.795214

**Published:** 2022-01-18

**Authors:** Limei Xiong, Hui Zhang, Yannan Guo, Yue Song, Yuhong Tao

**Affiliations:** ^1^ Division of Nephrology, Department of Pediatrics, West China Second University Hospital, Sichuan University, Chengdu, China; ^2^ Key Laboratory of Birth Defects and Related Diseases of Women and Children, Ministry of Education, Sichuan University, Chengdu, China; ^3^ Department of Pediatrics, Meishan Women and Children’s Hospital, Alliance Hospital of West China Second University Hospital, Sichuan University, Meishan, China

**Keywords:** vadadustat, chronic kidney disease, anemia, hypoxia-inducible factor prolyl hydroxylase inhibitor, iron utilization

## Abstract

**Background:** Vadadustat is a novel drug for treating anemia patients with chronic kidney disease (CKD), but its effect and safety remain uncertain. This study aimed to summarize the evidence for vadadustat in the treatment of CKD patients with anemia.

**Methods:** PubMed, Ovid Medline, Embase, Cochrane CENTRAL, Wanfang Data, China National Knowledge Infrastructure and an international trial register were searched from their inception to June 2021 for randomized controlled trials (RCTs) comparing the efficacy and safety of vadadustat to those of placebo or erythropoiesis-stimulating agents (ESAs) in treating anemia in CKD patients. Data were pooled in a meta-analysis, with results expressed as the mean difference for continuous outcomes and relative risk for categorical outcomes with 95% confidence intervals (95% CIs). The certainty of evidence was rated according to Cochrane methods and the GRADE approach.

**Results:** Ten RCTs comparing vadadustat with placebo (4 RCTs) or darbepoetin alfa (6 RCTs) were included (*n* = 8,438 participants). Compared with placebo, vadadustat increased the hemoglobin (Hb) response rate (risk ratio 5.27; 95% CI: 2.69 to 10.31; *p* < 0.001; high certainty of evidence) and Hb level from baseline (∆Hb) (mean difference (MD) 1.28; 95% CI: 0.83 to 1.73; *p* < 0.001; low certainty of evidence). Compared with placebo or darbepoetin alfa, vadadustat decreased hepcidin (MD -36.62; 95% CI: −54.95 to −18.30; *p* < 0.001) and ferritin (MD −56.24; 95% CI: −77.37 to −35.11; *p* < 0.001) levels and increased iron-binding capacity (MD 24.38; 95% CI: 13.69 to 35.07; *p* < 0.001), with a low to moderate certainty of evidence. Moderate to high certainty evidence suggested that compared with placebo or darbepoetin alfa, vadadustat significantly increased the risk of nausea and diarrhea but did not significantly increase the risk of serious adverse events, especially all-cause mortality, cardiac events and nonfatal stroke.

**Conclusion:** Vadadustat may safely improve Hb levels and promote iron utilization in CKD patients with anemia without increasing the incidence of serious adverse events.

## Introduction

Anemia is prevalent in patients with chronic kidney disease (CKD) (St Peter et al., 2018; Vestergaard et al., 2020; Wong et al., 2020; Hanna et al., 2021) and associated with an increased risk of progression of CKD, cardiovascular disease and mortality (Thorp et al., 2009; Fishbane and Spinowitz, 2018; Shaker et al., 2018; Palaka et al., 2020). Correcting anemia is vital for reducing mortality and hospitalization and improving the life quality of CKD patients. The current guidelines on anemia management of CKD mainly focus on the provision of recombinant human erythropoietin (EPO) and its analogs, known as erythropoiesis-stimulating agents (ESAs), along with iron supplementation. Although ESAs are effective in correcting anemia, their adverse events (AEs) cannot be neglected. Indeed, clinical data have shown that ESAs, such as darbepoetin alfa, are associated with an increased risk of cardiovascular events and death when supraphysiologic dosing of ESAs is administered or when targeting near-normal hemoglobin (Hb) levels (Parfrey et al., 2005; Singh et al., 2006; Pfeffer et al., 2009; Solomon et al., 2010). Therefore, alternative effective and safe therapeutic strategies are necessary for the treatment of renal anemia.

Hypoxia-inducible factor prolyl hydroxylase inhibitors (HIF-PHIs) promote erythropoiesis through the stimulation of endogenous EPO production, increasing uptake of iron and mobilization of iron stores, which can positively contribute to the correction and maintenance of Hb and iron metabolism (Provenzano et al., 2016; Haase, 2017; Sanghani and Haase, 2019). In addition, the increase in plasma EPO concentration of HIF-PHI-treated subjects is much lower than that of subjects treated with traditional ESAs when reaching target Hb, which may reduce cardiovascular risk and mortality (Holdstock et al., 2016; Parmar et al., 2019). Therefore, HIF-PHIs are being developed as a new therapy for anemia in patients with CKD (Babitt et al., 2021).

Vadadustat, also known as AKB-6548, is a novel oral HIF-PHI under investigation. Previous phase 2 trials have shown that vadadustat significantly elevates and maintains Hb concentrations compared with placebo in nondialysis-dependent CKD (NDD-CKD) patients (Pergola et al., 2016; Martin et al., 2017). Recently, multicenter randomized controlled trials (RCTs) on vadadustat versus placebo or an ESA in both dialysis-dependent (DD-CKD) and NDD-CKD patients have been completed (Nangaku et al., 2020; Nangaku et al., 2021a; Nangaku et al., 2021b; Chertow et al., 2021; Eckardt et al., 2021), but no rigorous systematic synthesis of all relevant data is available to date. To provide more evidence for vadadustat in the treatment of anemia in CKD, we incorporated phase 2 and 3 RCTs comparing vadadustat with placebo or ESAs and performed a systematic review to comprehensively evaluate the efficacy and safety of vadadustat for anemia in CKD patients compared with placebo or ESAs.

## Methods

We undertook and reported this systematic review and meta-analysis in accordance with the Preferred Reporting Items for Systematic Reviews and Meta-analyses (PRISMA) guidelines (Higgins et al., 2011). The protocol of this review was prospectively registered in International Prospective Register of Systematic Reviews (CRD42021273856).

### Data Sources and Search Strategy

We searched an international trial register (clinicaltrials.gov) and six electronic databases, including PubMed, Ovid Medline, EMBASE, Cochrane Central Register of Controlled Trials (CENTRAL), Wanfang Data and China National Knowledge Infrastructure (CNKI), for data from database inception to June 30, 2021. We searched relevant free-text words and medical subject headings that included all spellings of “chronic kidney disease”, “anemia”, “vadadustat” and “AKB-6548”. The corresponding key Chinese terms were used in Chinese databases. We selected publications without restrictions of origin, country or language. The reference lists of relevant review studies and included original studies were reviewed to identify additional relevant studies.

### Eligibility Criteria

Only RCTs evaluating the effect of vadadustat on patients with renal anemia were considered. The inclusion criteria were as follows: 1) adult CKD patients diagnosed with renal anemia with or without a need for dialysis; 2) vadadustat without dose or frequency restrictions as a treatment compared with placebo or ESAs; and 3) at least one of the primary or secondary outcome was available. If one cohort was reported in several publications, only the article with the largest sample size and longest duration was included.

### Outcome Measures

The primary outcomes were as follows:1) response rate of Hb: defined as an increase in Hb level of ≥1.0 g/dL from baseline or achievement of the target range (11.0–13.0 g/dl); 2) change in Hb level from baseline (∆Hb); 3) adverse events (AEs), which were defined as previously unobserved medical conditions, signs, and/or symptoms in the participant that emerged during the protocol-specified AE-reporting period, including signs or symptoms related to preexisting underlying disease, which were not present prior to the AE-reporting period; and 4) severe adverse events (SAEs), which were defined as death, life-threatening complications, events requiring urgent medical intervention or hospitalization (e.g., acute kidney injury, heart failure, cardiovascular events, vascular access thrombosis, severe infection), persistent or significant incapacitation or even disability, or any medically important event that did not meet the above criteria but could jeopardize a participant or require medical intervention or surgical intervention to prevent the occurrence of the above conditions.

The secondary outcomes were as follows: 1)major adverse events, including all-cause mortality, cardiac events and nonfatal stroke; 2) common adverse events, including gastrointestinal disorder (e.g., nausea, diarrhea, and vomiting), hypertension, hyperkalemia, nasopharyngitis, peripheral edema, hypotension, headache, fatigue and acute kidney injury; 3) changes in iron metabolism parameters, including hepcidin, ferritin, total iron-binding capacity (TIBC); and 4) use of rescue medication, including rescue with red blood cell (RBC) transfusion or ESAs(except darbepoetin alfa) during the treatment period.

### Data Collection

Two investigators independently screened the titles and abstracts or read the full text to identify eligible studies according to the predefined eligibility criteria. Another two researchers extracted data and assessed the risk of bias in duplicate. Data extraction was performed using a self-designed data extraction sheet. The main clinical characteristics were year of publication, authors, clinical trial number, type of study, population characteristics, sample size, vadadustat dosage, treatment duration, control treatment, and primary and secondary outcomes. Any discrepancies were resolved by discussion with a senior investigator. The risk of bias of each RCT was assessed using the Cochrane risk of bias tool. We also attempted to contact authors by email to obtain missing data for some of the included trials.

### Data Analysis

For continuous outcomes, mean differences (MDs) or standardized mean differences (SMDs) were calculated with the inverse variance method. For dichotomous outcomes, risk ratios (RRs) and random-effects models were used for analysis. We calculated the 95% CI for each effect size estimate. Heterogeneity between studies was estimated by the *I* ^2^ statistic and considered low if *I* ^2^ < 50% (Higgins and Thompson, 2002). Subgroup analyses were performed according to interventions of the control group and stratified by whether the patients were on dialysis, the duration of trial, and the dosage of vadadustat when possible. *p* < 0.05 was indicative of statistical significance. Sensitivity analyses were performed to test the robustness of the findings by removing studies rated as having a “high” risk of bias. The potential publication bias of primary outcomes was estimated by Begg’s test and Egger’s test.

Review Manager (RevMan, Version 5.3, The Nordic Cochrane Center, Cochrane Collaboration, Copenhagen, Denmark) and STATA 12.0 (StatsDirect Ltd., Cambridge, United Kingdom) were used to perform statistical analysis. The quality of evidence was estimated by the Grading of Recommendation Assessment, Development and Evaluation (GRADE) method on the basis of five domains: risk of bias, consistency, directness, precision, and publication bias. The certainty of evidence was classified into high, moderate, low and very low levels.

## Results

### Literature Search

**FIGURE 1:**
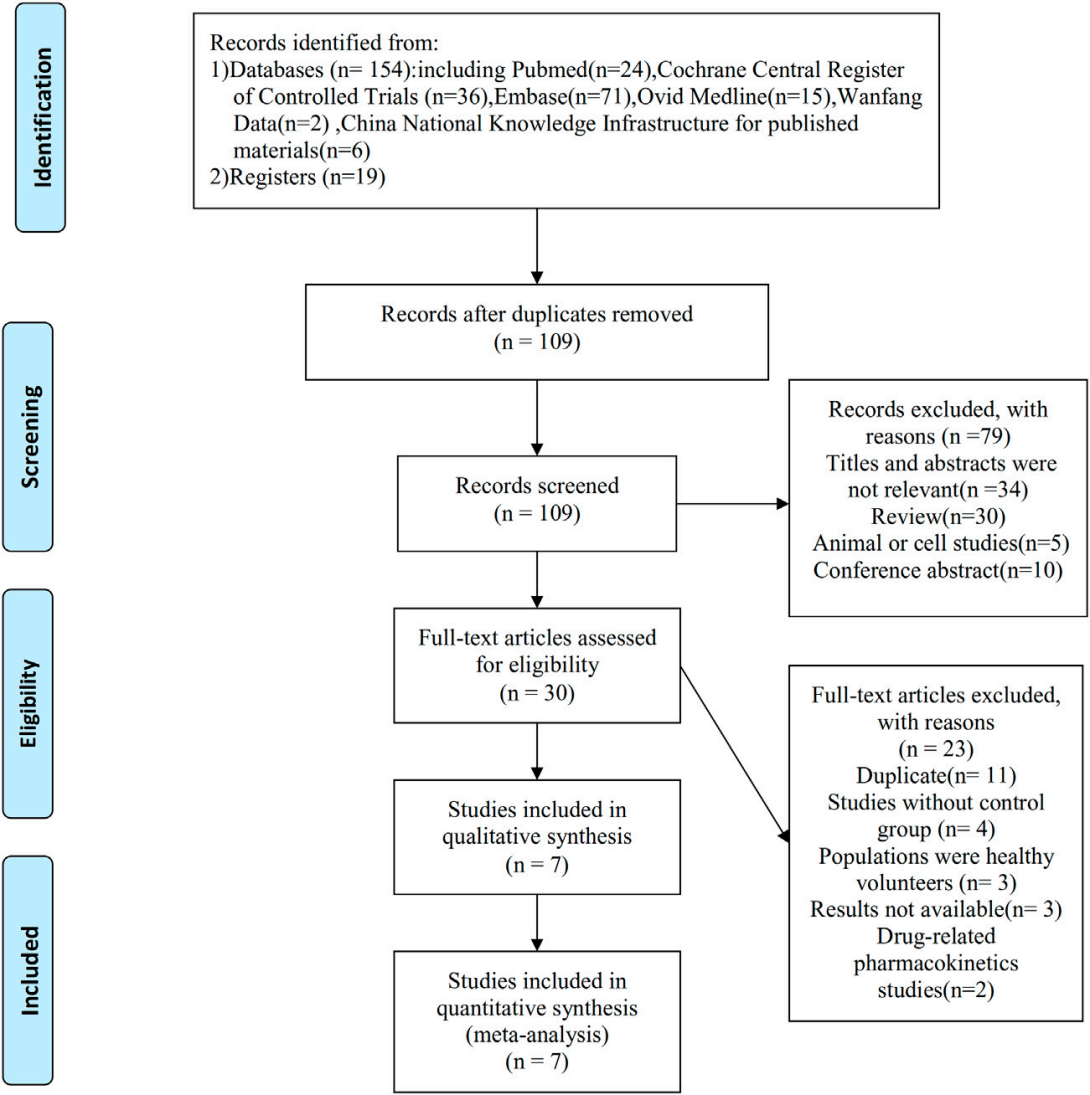
Flowchart demonstrating the process of study selection.

A total of 173 records were obtained according to the initial search strategy, of which 19 relevant studies were found in the international trial register. After removing 64 duplicate records, a total of 90 records were found through the database search. We screened 109 records and obtained 30 records to assess eligibility. Of these, 23 were excluded, most often because the records found in the international trial register were regarded as duplicate records of available published papers (*n* = 11) or the study had an ineligible design (*n* = 4). Finally, 7 articles that included 10 RCTs were selected for analysis (Pergola et al., 2016; Martin et al., 2017; Nangaku et al., 2020; Chertow et al., 2021; Eckardt et al., 2021; Nangaku et al., 2021a; Nangaku et al., 2021b) (Figure 1).

### Characteristics of Included Studies

Ten RCTs from the 7 studies included in the systematic review were conducted in different regions worldwide, including the United States, Europe, Japan and other places. The characteristics of each trial are summarized in Table 1. A total of 8,438 participants diagnosed with CKD-related anemia were enrolled. Of the 10 trials, 6 investigated the effect and safety of vadadustat versus placebo/ESA (darbepoetin alfa) for the treatment of anemia in NDD-CKD subjects (Pergola et al., 2016; Martin et al., 2017; Nangaku et al., 2020; Chertow et al., 2021; Nangaku et al., 2021a). Another 4 trials investigated the effect and safety of vadadustat versus placebo/ESA (darbepoetin alfa) for the treatment of anemia in DD-CKD subjects (Nangaku et al., 2020; Eckardt et al., 2021; Nangaku et al., 2021b). Participants in the control group received placebo in 4 trials and darbepoetin alfa in 6 trials. The follow-up duration ranged from 6 to 52 weeks. The mean age of the intervention subjects ranged from 56.5 to 71.7 years and that of the control subjects ranged from 55.6 to 72.2 years. In all trials, the demographic characteristics of the intervention and control groups were similar at baseline. However, doses of vadadustat differed among the studies. ∆Hb and AEs were the primary endpoints for all eligible trials.

**TABLE 1:** Characteristics of included trials.

Authors (study year)	Study design	Registration number	Diagnosis of patients	Duration of treatment	Intervention (dosage)	Control	Sample size (intervention/control)	Main outcome measurement
Pergola et al. (2016)	RCT	NCT01906489	NDD-CKD	20 weeks	Vadadustat (150–600 mg)	Placebo	210 (138/72)	Hb response, ∆Hb, iron metabolism parameters, AEs, SAEs, rescue therapy
Martin et al. (2017)	RCT	NCT01381094	NDD-CKD	6 weeks	Vadadustat (240,370,500,630 mg)	Placebo	91 (72/19)	Hb response, ∆Hb, iron metabolism parameters, AEs, SAEs
Nangaku et al. (2020)	RCT	NCT03054337	NDD-CKD	16 weeks^a^	Vadadustat (150,300,600 mg)	Placebo	51 (37/14)	∆Hb, iron metabolism parameters, AEs, SAEs, rescue therapy
	RCT	NCT03054350	DD-CKD	16 weeks^a^	Vadadustat (150,300,600 mg)	Placebo	60 (45/15)	∆Hb, iron metabolism parameters, AEs, SAEs, rescue therapy
Chertow et al. (2021)	Open-label RCT	NCT02648347	NDD-CKD (ESA-untreated patients)	52 weeks	Vadadustat (150∼600 mg)	Darbepoetin alfa	1751 (879/872)	Hb response, ∆Hb, AEs, SAEs, rescue therapy
	Open-label RCT	NCT02680574	NDD-CKD (ESA-treated patients)	52 weeks	Vadadustat (150∼600 mg)	Darbepoetin alfa	1725 (862/863)	Hb response, ∆Hb, AEs, SAEs, rescue therapy
Eckardt et al. (2021)	Open-label RCT	NCT02865850	Incident DD-CKD^b^	52 weeks	Vadadustat (150∼600 mg)	Darbepoetin alfa	369 (181/188)	Hb response, ∆Hb, iron metabolism parameters, AEs, SAEs, rescue therapy
	Open-label RCT	NCT02892149	Prevalent DD-CKD^c^	52 weeks	Vadadustat (150∼600 mg)	Darbepoetin alfa	3,554 (1777/1777)	Hb response, ∆Hb, iron metabolism parameters, AEs, SAEs, rescue therapy
Nangaku et al. (2021a)	Open-label RCT	NCT03329196	NDD-CKD	52 weeks	Vadadustat (150∼600 mg)	Darbepoetin alfa	304 (151/153)	Hb response, ∆Hb, iron metabolism parameters, AEs, SAEs
Nangaku et al. (2021b)	RCT	NCT03439137	DD-CKD	52 weeks	Vadadustat (150∼600 mg)	Darbepoetin alfa	323 (162/161)	Hb response, ∆Hb, iron metabolism parameters, AEs, SAEs

Abbreviations: CKD, chronic kidney disease; RCT, randomized controlled trial; NDD-CKD, nondialysis-dependent chronic kidney disease; DD-CKD, dialysis-dependent chronic kidney disease; ESAs, erythropoiesis-stimulating agents; ∆Hb, change in Hb level from baseline; AEs, adverse events; SAEs, serious adverse events.

a 6 weeks of fixed vadadustat doses, but over the next 10 weeks, placebo patients switched to vadadustat 150, 300 or 600 mg.

b Incident DD-CKD, patients were to have initiated dialysis within 16 weeks before screening and were to have had limited exposure to ESAs.

c Prevalent DD-CKD, patients were undergoing maintenance dialysis for at least 12 weeks before screening and were to have been receiving treatment with an ESA.

### Risk-of-Bias Assessment

The risk of bias of the 10 trials was assessed according to the Cochrane Handbook. All trials inadequately reported randomization, allocation concealment and blinding of outcome assessment, which was regarded as having a low risk of bias. Five trials in 3 studies had an unclear risk of bias related to participant and personnel blinding because an open-label design carries an inherent bias that might affect AE reporting (Chertow et al., 2021; Eckardt et al., 2021; Nangaku et al., 2021a). In terms of incomplete outcome data, 3 trials had a high risk of bias because missing data were not balanced between the intervention group and the control group and missing outcomes differed (Pergola et al., 2016; Nangaku et al., 2021a; Nangaku et al., 2021b). Nine trials had a low risk of bias in selective reporting, and only one have a high risk of bias (Pergola et al., 2016). Other risks, including potential sources of bias, were not found in any of the included studies (Supplementary Figure S1).

### Meta-Analysis of Primary Outcomes

#### Response Rate of Hb

Eight trials reported the response rate of Hb (Pergola et al., 2016; Martin et al., 2017; Nangaku et al., 2021a; Nangaku et al., 2021b; Chertow et al., 2021; Eckardt et al., 2021). Meta-analysis,with a total of 8,127 participants, revealedno significant difference between the vadadustat and control groups (RR 1.0; 95% CI: 0.90 to 1.12; *p* = 0.96; *I* ^2^ = 83%), with high heterogeneity (low certainty of evidence). Sensitivity analysis showed a similar result (RR 1.0; 95% CI: 0.90 to 1.11; *p* = 0.99; *I* ^2^ = 79%).

**FIGURE 2:**
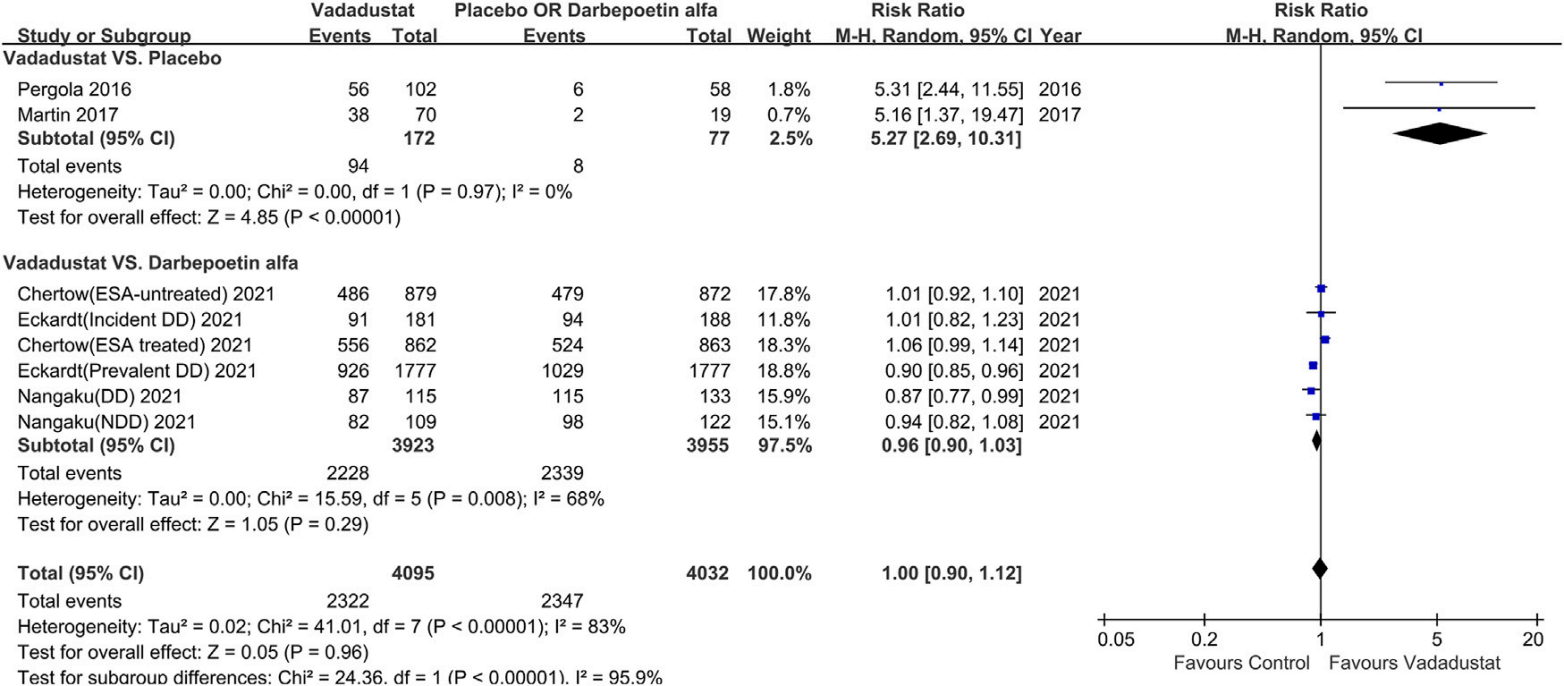
Risk ratios of the Hb response rate for vadadustat versus placebo and darbepoetin alfa. Hb, hemoglobin. The registration number of Chertow (ESA-untreated) 2021 corresponds to NCT02648347; Chertow (ESA treated) 2021 corresponds to NCT02680574, Eckardt (incident DD) 2021 corresponds to NCT02865850, Eckardt (prevalent DD)2021 corresponds to NCT02892149, Nangaku (NDD) 2021 corresponds to NCT03329196, Nangaku (DD) 2021 corresponds to NCT03439137.

Based on subgroup analysis of placebo-controlled trials, the response rate of Hb was significantly higher in the vadadustat group than in the placebo group (RR 5.27; 95% CI: 2.69 to 10.31; *p* < 0.001; *I* ^2^ = 0%; high certainty of evidence; Figure 2), with low heterogeneity. Subgroup analysis of darbepoetin alfa-controlled trials showed no significant differences between the vadadustat and darbepoetin alfa groups (RR 0.96; 95% CI: 0.90 to 1.03; *p* = 0.29; *I* ^2^ = 68%; low certainty of evidence; Figure 2). Other subgroup analyses are presented in Table 2.

**TABLE 2:** Subgroup analysis of the response rate of Hb and ∆Hb.

Effect on Hb	Subgroup analysis^a^		Number of studies	Number of patients	Risk ratio (95%CI)/Mean difference (95%CI)	*I* ^*2*^
Response rate of Hb	Vadadustat vs. placebo	Dialysis status				
		NDD-CKD	2 RCTs	249 participants	5.27 (2.69,10.31)	0%
		Follow up weeks				
		<24 weeks	2 RCTs	249 participants	5.27 (2.69,10.31)	0%
	Vadadustat vs.darbepoetin alfa	Dialysis status				
		NDD-CKD	3 RCTs	3,707 participants	1.02 (0.96,1.08)	27%
		DD-CKD	3 RCTs	4,171 participants	0.90 (0.86,0.95)	0%
		Follow up weeks				
		≥24 weeks	6 RCTs	7,878 participants	0.96 (0.90,1.03)	68%
∆Hb	Vadadustat vs.placebo	Dialysis status				
		NDD-CKD	3 RCTs	264 participants	1.25 (0.76, 1.73)	89%
		DD-CKD	1 RCT	43 participants	1.56 (0.51, 2.61)	—
		Follow up weeks				
		<24 weeks	4 RCTs	307 participants	1.28 (0.83, 1.73)	84%
	Vadadustat vs.darbepoetin alfa	Dialysis status				
		NDD-CKD	1 RCT	304 participants	−0.13 (−0.35,0.09)	—
		DD-CKD	1 RCT	322 participants	−0.39 (−0.57, −0.21)	—
		Follow up weeks				
		≥24 weeks	2 RCTs	626 participants	−0.27 (−0.52, −0.01)	68%

Abbreviations: RCTs, randomized controlled trials; NDD-CKD, nondialysis-dependent chronic kidney disease; DD-CKD, dialysis-dependent chronic kidney disease; ∆Hb, change in Hb level from baseline; “-”, not estimable.

a Subgroup analysis was conducted within placebo-controlled trials and darbepoetin alfa controlled trials, and stratified by dialysis status and follow-up duration.

#### Change in Hb Level From Baseline(∆Hb)

Ten trials reported ∆Hb (unit: g/dL). Of these, 4 reported ∆Hb by the least-squares mean (±SE), which could not be included in the meta-analysis (Chertow et al., 2021; Eckardt et al., 2021). One trial provided only raw data of one group (vadadustat 630 mg versus placebo) (Martin et al., 2017). Therefore, 6 trials that included 933 participants were pooled; of these, the control group of 4 trials received placebo (Pergola et al., 2016; Martin et al., 2017; Nangaku et al., 2020), and that of 2 trials received darbepoetin alfa (Nangaku et al., 2021a; Nangaku et al., 2021b). Meta-analysis showed that the vadadustat group exhibited a significant increase in Hb levels compared with the control group (MD 0.76; 95% CI: 0.11 to 1.40; *p =* 0.02; *I* ^2^ = 98%; low certainty of evidence), with high heterogeneity. Sensitivity analysis showed a similar effect (MD 1.48; 95% CI: 1.22 to 1.73; *p <* 0.001; *I* ^2^ = 0%), with very low heterogeneity after excluding the study with a high risk of bias, and the remaining trials were placebo-controlled (Martin et al., 2017; Nangaku et al., 2020).

**FIGURE 3:**
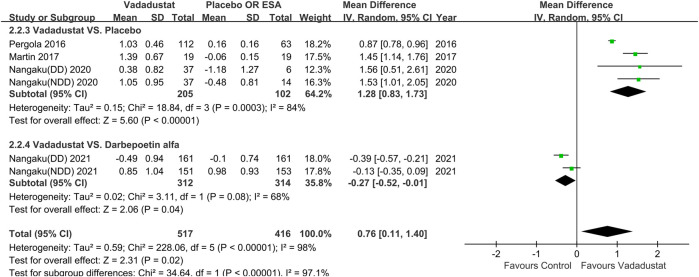
Change in Hb level from baseline for vadadustat versus placebo and darbepoetin alfa. Hb, hemoglobin; ESA, erythropoiesis-stimulating agent, only darbepoetin alfa. The registration number of Nangaku (NDD) 2020 corresponds to NCT03054337; Nangaku (DD) 2020 corresponds to NCT03054350, Nangaku (NDD) 2021 corresponds to NCT03329196, Nangaku (DD) 2021 corresponds to NCT03439137.

In subgroup analysis of placebo-controlled trials, ∆Hb was significantly higher in the vadadustat group than in the placebo group (MD 1.28; 95% CI: 0.83 to 1.73; *p* < 0.001; *I* ^2^ = 84%; low certainty of evidence; Figure 3), with high heterogeneity. In subgroup analysis among darbepoetin alfa-controlled trials, ∆Hb was significantly lower in the vadadustat group than in the darbepoetin alfa group (MD -0.27; 95% CI: −0.52 to −0.01; *p =* 0.04; *I* ^2^ = 68%; moderate certainty of evidence; Figure 3). Other subgroup analyses are provided in Table 2.

#### Incidence of AEs

**FIGURE 4:**
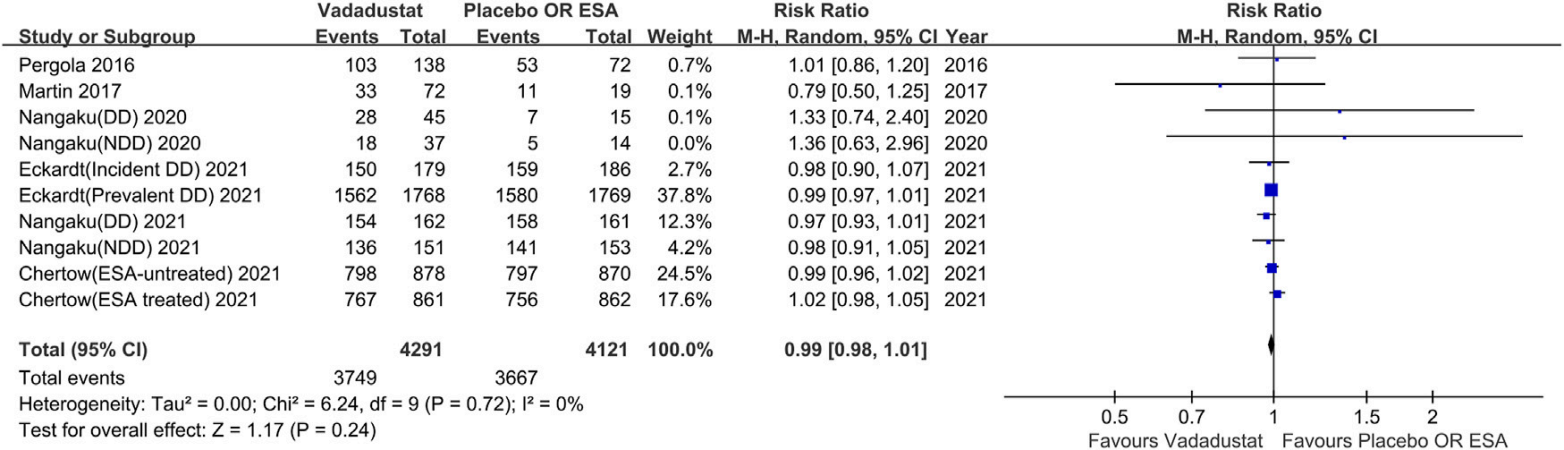
Any adverse events with vadadustat versus placebo or darbepoetin alfa. EAS, erythropoiesis-stimulating agent, only darbepoetin alfa. The registration number of Nangaku (NDD) 2020 corresponds to NCT03054337, Nangaku (DD) 2020 corresponds to NCT03054350, Chertow (ESA-untreated) 2021 corresponds to NCT02648347, Chertow (ESA treated) 2021 corresponds to NCT02680574, Eckardt (incident DD) 2021 corresponds to NCT02865850, Eckardt (prevalent DD) 2021 corresponds to NCT02892149, Nangaku (NDD) 2021 corresponds to NCT03329196, Nangaku (DD) 2021 corresponds to NCT03439137.

Ten trials reported the incidence of any AEs during the trial. Meta-analysis of a total of 8,412 participants indicated that vadadustat did not significantly increase the risk of any AEs compared with the control (RR 0.99; 95% CI: 0.98 to 1.01; *p* = 0.24; *I* ^2^ = 0%; high certainty of evidence; Figure 4), with low heterogeneity. A similar finding was obtained insensitivity analysis (RR 1.0; 95% CI: 0.98 to 1.01; *p* = 0.57; *I* ^2^ = 0%).

Subgroup analysis based on whether the patients were on dialysis, the intervention used in the control group, and the duration of trial also revealed no significant difference in the incidence of AEs between the vadadustat group and control group.

#### Incidence of SAEs

**FIGURE 5:**
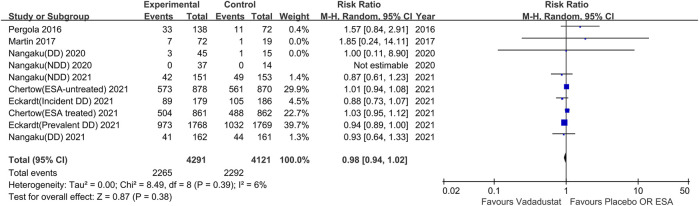
Serious adverse events with vadadustat versus placebo or darbepoetin alfa. The registration number of Nangaku (NDD) 2020 corresponds to NCT03054337; Nangaku (DD)2020 corresponds to NCT03054350, Chertow (ESA-untreated) 2021 corresponds to NCT02648347, Chertow (ESA treated) 2021 corresponds to NCT02680574, Eckardt (incident DD)2021 corresponds to NCT02865850, Eckardt (prevalent DD) 2021 corresponds to NCT02892149, Nangaku (NDD) 2021 corresponds to NCT03329196, Nangaku (DD) 2021 corresponds to NCT03439137.

Ten trials reported the incidence of SAEs. Meta-analysis, with a total of 8,412 participants, showed that vadadustat did not significantly increase the risk of SAEs compared with the control (RR 0.98; 95% CI: 0.94 to 1.02; *p* = 0.38; *I* ^2^ = 6%; high certainty of evidence; Figure 5), with low heterogeneity. Sensitivity analysis showed a similar effect (RR 0.98; 95% CI: 0.94 to 1.03; *p =* 0.44; *I* ^2^ = 13%).

Subgroup analysis of dialysis-dependent participants demonstrated that vadadustat could decrease the risk of SAEs compared with placebo or darbepoetin alfa (RR 0.94; 95% CI: 0.89 to 0.99; *p* = 0.02; *I* ^2^ = 0%), with low heterogeneity. Other subgroup analyses based on interventions in the control group, the duration of the trial and the nondialysis-dependent population showed no significant difference in the incidence of SAEs between the vadadustat group and control group.

### Meta-Analysis of Secondary Outcomes

#### Major Adverse Events

The incidence of all-cause mortality and cardiac events, including cardiac death, cardiac arrest, and nonfatal myocardial infarction, was reported in ten trials, with a total of 8,412 participants. Four trials, with a total of 7,373 participants, reported nonfatal stroke (Chertow et al., 2021; Eckardt et al., 2021). Chertow et al. (Chertow et al., 2021) and Eckardt et al. (Eckardt et al., 2021) reported pooled data from 2 RCTs in their study because of the same study design. No significant difference in the incidence of all-cause mortality (RR 1.0; 95% CI: 0.9 to 1.11; *p =* 0.99; *I* ^2^ = 0%; high certainty of evidence; Supplementary Figure S2A), cardiac events (RR 1.03; 95% CI: 0.88 to 1.20; *p* = 0.74; *I* ^2^ = 0%; high certainty of evidence; Supplementary Figure S2B) or nonfatal stroke (RR 0.92; 95% CI: 0.55 to 1.57; *p =* 0.77; *I* ^2^ = 56%; moderate certainty of evidence; Supplementary Figure S2C)was found between the vadadustat group and control group. Sensitivity analyses also indicated no significant differences between the two groups with regard to the incidence of the above major adverse events. Subgroup analysis stratified according to interventions of the control group, dialysis status and the duration of the trial also revealed no significant difference between the two groups.

#### Common Adverse Events

Only the incidence rates of nausea, diarrhea and hyperkalemia differed significantly between the vadadustat group and control group. Regarding the incidence rates of other common adverse events, there was no significant difference between the two groups (Table 3).

**TABLE 3:** Analysis of common adverse events.

Adverse events	Number of trials	Number of patients	Risk ratio (95%CI)	*I* ^2^ (%)
Gastrointestinal manifestations				
Nausea	8 RCTs	8,017 participants	1.21 (1.04,1.40)	0
Vomiting	5 RCTs	4,589 participants	1.06 (0.66,1.72)	51
Diarrhea	7 RCTs	7,966 participants	1.35 (1.19,1.53)	0
Hypertension	7 RCTs	7,788 participants	0.86 (0.71,1.05)	53
Hyperkalemia	8 RCTs	8,261 participants	0.84 (0.74,0.96)	0
Nasopharyngitis	7 RCTs	8,060 participants	1.00 (0.87,1.15)	0
Edema, peripheral	7 RCTs	7,938 participants	1.06 (0.87,1.28)	27
Hypotension	6 RCTs	7,634 participants	1.02 (0.82,1.26)	16
Acute kidney injury	5 RCTs	4,036 participants	1.10 (0.87,1.39)	0
Headache	7 RCTs	8,060 participants	1.02 (0.71,1.47)	56
Fatigue	4 RCTs	3,732 participants	0.85 (0.60,1.19)	0

Abbreviations: RCTs, randomized controlled trials.

The incidence of nausea (RR 1.21; 95% CI: 1.04 to 1.40; *p* = 0.01; *I* ^2^ = 0%; high certainty of evidence; Supplementary Figure S3) and diarrhea (RR 1.35; 95% CI: 1.19 to 1.53; *p* < 0.001; *I* ^2^ = 0%; high certainty of evidence; Supplementary Figure S4) in the vadadustat-treated population was significantly higher than that in the control group, with low heterogeneity. Based on subgroup analysis of placebo-controlled trials, there was no significant difference in the incidence of nausea (RR 2.25; 95% CI: 0.78 to 6.47; *p* = 0.13; *I* ^2^ = 0%) or diarrhea (RR 2.10; 95% CI: 0.73 to 6.01; *p* = 0.17; *I* ^2^ = 0%) between the two groups. In subgroup analysis of darbepoetin alfa-controlled trials, the incidence rates of nausea (RR 1.19; 95% CI: 1.02 to 1.39; *p =* 0.03; *I* ^2^ = 0%) and diarrhea (RR 1.34; 95% CI: 1.18 to 1.52; *p <* 0.001; *I* ^2^ = 0%) were significantly higher in the vadadustat group than darbepoetin alfa group.

The incidence of hyperkalemia in the vadadustat-treated population was significantly lower than that in the control group (RR 0.84; 95% CI: 0.74 to 0.96; *p =* 0.01; *I* ^2^ = 0%; high certainty of evidence; Supplementary Figure S5), with low heterogeneity. In subgroup analysis among placebo-controlled trials, no significant difference in the incidence of hyperkalemia between the two groups was detected (RR 3.35; 95% CI: 0.41 to 27.69; *p =* 0.26; *I* ^2^ = 0%). Nonetheless, the incidence of hyperkalemia was significantly lower in the vadadustat group than in the darbepoetin alfa groupaccording to subgroup analysis of darbepoetin alfa-controlled trials (RR 0.84; 95% CI: 0.73 to 0.96; *p <* 0.001; *I* ^2^ = 0%).

#### Changes in Iron Metabolism Parameters

Eight trials reported hepcidin and ferritin, but the original data were not provided in 2 trials (Nangaku et al., 2021a; Nangaku et al., 2021b). Meta-analysis of 6 RCTs that pooled 4,314 participants showed a significant reduction in hepcidin (MD −36.62; 95% CI: −54.95 to −18.30; *p <* 0.001; *I* ^2^ = 84%; moderate certainty of evidence; Supplementary Figure S6A) and ferritin (MD −56.24; 95% CI: −77.37 to −35.11; *p <* 0.001; *I* ^2^ = 53%; moderate certainty of evidence; Supplementary Figure S6B) levels in subjects treated with vadadustat compared with placebo or darbepoetin alfa (Pergola et al., 2016; Martin et al., 2017; Nangaku et al., 2020; Eckardt et al., 2021). Sensitivity analysis also indicated a similar reduction in hepcidin (MD −32.51; 95% CI: −51.78 to −13.25; *p <* 0.001; *I* ^2^ = 85%) and ferritin (MD -54.91; 95% CI: −79.74 to −30.08; *p <* 0.001; *I* ^2^ = 62%) levels. Six RCTs reported TIBC, but the original data from 2 trials were unavailable (Nangaku et al., 2021a; Nangaku et al., 2021b). Meta-analysis of 4 RCTs including 393 participants demonstrated an increase in TIBC in the vadadustat group compared with the control group (MD 24.38; 95% CI: 13.69 to 35.07; *p <* 0.001; *I* ^2^ = 96%; low certainty of evidence; Supplementary Figure S6C), with high heterogeneity (Pergola et al., 2016; Martin et al., 2017; Nangaku et al., 2020). Sensitivity analysis showed a similar increase (MD 38.94; 95% CI: 2.47 to 75.40; *p =* 0.04; *I* ^2^ = 97%). Subgroup analyses stratified according to interventions in the control group, dialysis status and the duration of the trial are provided in Table 4.

**TABLE 4:** Subgroup analysis of iron metabolism parameters.

Parameter	Subgroup analysis^a^		Number of studies	Number of patients	Mean difference (95%CI)	*I* ^2^
Hepcidin	Vadadustat vs. placebo	Dialysis status				
		NDD-CKD	3 RCTs	348 participants	−43.00 (−66.33, −19.68)	45%
		DD-CKD	1 RCT	43 participants	−66.59 (-83.99, −49.19)	—
		Follow up weeks				
		<24 weeks	4 RCTs	391 participants	−51.90 (−75.97, −27.84)	75%
	Vadadustat vs.darbepoetin alfa	Dialysis status				
		DD-CKD	2 RCTs	3923participants	−14.52 (−37.14, 8.10)	77%
		Follow up weeks				
		≥24 weeks	2 RCTs	3923participants	−14.52 (−37.14, 8.10)	77%
Ferritin	Vadadustat vs.placebo	Dialysis status				
		NDD-CKD	3 RCTs	348 participants	−57.89 (−74.59, −41.19)	0%
		DD-CKD	1 RCT	43 participants	−88.69 (−120.53, −56.85)	—
		Follow up weeks				
		<24 weeks	4 RCTs	391 participants	−64.54 (−79.32, −49.75)	0%
	Vadadustat vs.darbepoetin alfa	Dialysis status				
		DD-CKD	2 RCTs	3,923 participants	−4.33 (−103.08, 94.41)	78%
		Follow up weeks				
		≥24 weeks	2 RCTs	3,923 participants	−4.33 (−103.08, 94.41)	78%
TIBC	Vadadustat vs.placebo	Dialysis status				
		NDD-CKD	3 RCTs	350 participants	15.73 (6.33, 25.13)	95%
		DD-CKD	1 RCT	43 participants	46.80 (33.46, 60.14)	—
		Follow up weeks				
		<24 weeks	4 RCTs	393 participants	24.38 (13.69, 35.07)	96%

Abbreviations: RCT, randomized controlled trial; NDD-CKD, nondialysis-dependent chronic kidney disease; DD-CKD, dialysis-dependent chronic kidney disease; TIBC, total iron-binding capacity.

a Subgroup analysis was conducted within placebo-controlled trials and darbepoetin alfa-controlled trials and stratified by dialysis status and follow-up duration.

#### Use of Rescue Therapy

Seven trials comprising 6,850 participants reported the use of ESA rescue medication and RBC transfusion (Pergola et al., 2016; Nangaku et al., 2020; Chertow et al., 2021; Eckardt et al., 2021). No significant difference between vadadustat and control groups was found in the proportion of patients requiring ESA rescue (RR 0.56; 95% CI: 0.3 to 1.04; *p =* 0.07; *I* ^2^ = 92%; moderate certainty of evidence; Supplementary Figure S7A) or RBC transfusion (RR 0.99; 95% CI: 0.62 to 1.58; *p =* 0.96; *I* ^2^ = 24%; high certainty of evidence; Supplementary Figure S7B). Subgroup analysis showed that vadadustat induced a significant reduction in the use of ESA rescue (RR 0.27; 95% CI: 0.14 to 0.5; *p <* 0.001; *I* ^2^ = 0%) and RBC transfusion (RR 0.12; 95% CI: 0.02 to 0.74; *p =* 0.02; *I* ^2^ = 0%), compared with placebo. However, the differences in ESA rescue (RR 0.75; 95% CI: 0.37 to 1.49; *p =* 0.41; *I* ^2^ = 94%) and RBC transfusion (RR 1.11; 95% CI: 0.77 to 1.60; *p =* 0.56; *I* ^2^ = 0%) were nonsignificant between vadadustat and darbepoetin alfa. Other subgroup analyses are given in Table 5.

**TABLE 5:** Subgroup analysis of ESA rescue and RBC transfusion.

Rescue therapy	Subgroup analysis^a^		Number of studies	Number of patients	Risk ratio (95%CI)	*I* ^2^
ESA rescue	Vadadustat vs.placebo	Dialysis status				
		NDD-CKD	2 RCTs	261 participants	0.26 (0.1, 0.67)	—
		DD-CKD	1 RCT	59 participants	0.27 (0.12, 0.62)	—
		Follow up weeks				
		<24 weeks	3 RCTs	320 participants	0.27 (0.14, 0.50)	0%
	Vadadustat vs.darbepoetin alfa	Dialysis status				
		NDD-CKD	2 RCTs	3,473 participants	0.41 (0.31, 0.55)	0%
		DD-CKD	2 RCTs	3,057 participants	1.21 (1.05, 1.41)	0%
		Follow up weeks				
		≥24 weeks	4 RCTs	6530participants	0.75 (0.37, 1.49)	94%
RBC transfusion	Vadadustat vs.placebo	Dialysis status				
		NDD-CKD	2 RCTs	261 participants	0.18 (0.01, 4.24)	—
		DD-CKD	1 RCT	59 participants	0.10 (0.01, 0.92)	—
		Follow up weeks				
		<24 weeks	3 RCTs	320 participants	0.12 (0.02, 0.74)	0%
	Vadadustat vs.darbepoetin alfa	Dialysis status				
		NDD-CKD	2 RCT	3,473 participants	1.11 (0.66, 1.85)	0%
		DD-CKD	2 RCTs	3,057 participants	1.12 (0.67, 1.88)	0%
		Follow up weeks				
		≥24 weeks	4 RCTs	6,530 participants	1.11 (0.77, 1.60)	0%

Abbreviations: RCT, randomized controlled trial; NDD-CKD, nondialysis-dependent chronic kidney disease; DD-CKD, dialysis-dependent chronic kidney disease; EAS, erythropoiesis-stimulating agent.

a subgroup analysis was conducted within placebo-controlled trials and darbepoetin alfa-controlled trials, and stratified by dialysis status and follow-up duration.

### Publication Bias

Egger’s test (*p =* 0.20) and Begg’s test (*p =* 0.39) for the incidence of Hb response, Egger’s test (*p =* 0.86) and Begg’s test (*p =* 0.59) for AEs and Egger’s test (*p =* 0.67) and Begg’s test (*p =* 0.47) for SAEs suggested no statistically significant publication bias. However, Egger’s test (*p =* 0.02) and Begg’s test (*p =* 0.26) suggested that there might be publication bias for ∆Hb.

## Discussion

This systematic review and meta-analysis were designed to comprehensively evaluate the efficacy and safety of vadadustat in CKD patients with anemia. The major results were as follows. First, vadadustat showed more favorable effects than placebo regarding increased Hb levels and Hb response rates and a reduction in the use of rescue therapy. Second, vadadustat significantly decreased serum ferritin and hepcidin levels and increased TIBC, with low to moderate certainty of evidence compared with placebo or darbepoetin alfa. Third, there was moderate to high certainty of evidence showing that the proportion of SAEs, especially major adverse events including all-cause mortality, cardiac events and nonfatal stroke, was similar in the vadadustat and control groups (placebo or darbepoetin alfa). However, vadadustat significantly increased the incidence rates of nausea and diarrhea compared with the control treatment.

A recent network meta-analysis showed that various HIF-PHIs, including roxadustat, daprodustat, molidustat, desidustat, and enarodustat, have significant therapeutic effects on anemia in patients not on dialysis, similar to ESAs (Zheng et al., 2020). In our study, the efficacy of vadadustat in terms of anemia correction agreed with other kinds of HIF-PHIs compared with placebo in NDD-CKD patients (Jia et al., 2019; Liu et al., 2020; Wang et al., 2020). Furthermore, we found that vadadustat was noninferior to darbepoetin alfa in the NDD-CKD population, and no significant difference in ∆Hb and Hb response rate was observed between the two groups. This work might provide alternatives for NDD-CKD patients who are unwilling to undergo ESA treatment or for whom ESAs are not effective. However, in the DD-CKD population, a significant difference in ∆Hb (MD −0.39; 95% CI: −0.57 to −0.21; *p <* 0.001) was observed, showing that darbepoetin alfa might be more effective than vadadustat. Nevertheless, this result must be interpreted with caution because it was based on a small sample and contradicts other high-quality trials. In the present report, two high-quality multicenter RCTs enrolling more than 3,000 participants were not included in the meta-analysis because ∆Hb was not described as the mean (±SD). The two studies demonstrated that vadadustat met the prespecified noninferiority criterion for hematologic efficacy compared with darbepoetin alfa, which was in contrast to our analysis. Thus, more studies are needed to optimize the effect of vadadustat compared with ESAs for DD-CKD patients. Moreover, neither of the placebo-controlled studies included involved long-term follow-up (≥24 weeks); thus, we were unable to estimate the beneficial effect of vadadustat in CKD patients beyond 24 weeks. Accordingly, more studies incorporating long-term follow-up are needed.

Measuring iron metabolism is critical for iron supplement therapy in CKD patients with anemia. Excess hepcidin is considered to be a contributing factor to anemia in CKD patients, as it impairs iron absorption from the diet and iron mobilization from body stores, which is suppressed by activation of the HIF pathway (Babitt and Lin, 2010; Batchelor et al., 2020). In our study, vadadustat significantly decreased ferritin and hepcidin levels and increased TIBC, with high heterogeneity. Therefore, we performed subgroup analysis and found that vadadustat better promoted iron utilization in CKD patients than placebo, which was consistent with other meta-analyses (Li et al., 2021). Among darbepoetin alfa-controlled trials, vadadustat also exhibited a tendency to decrease hepcidin and ferritin levels in DD-CKD patients, but the differences were not statistically significant. Indeed, there were some disagreements regarding the effect of HIF-PHIs in iron metabolism parameters compared with traditional ESAs (Volke et al., 2009; Liu et al., 2012; Mastrogiannaki et al., 2012). For example, Wen et al.‘s study, which analyzed 19 studies, reported a significant reduction in hepcidin and ferritin levels with HIF-PHIs compared with ESAs in DD-CKD patients (Wen et al., 2020). In contrast, Wang et al.‘s study, which included 26 trials, reported no significant reduction in hepcidin levels with HIF-PHIs compared with ESAs in DD-CKD patients (Wang et al., 2020). These inconsistent conclusions might be attributable to various iron supplements as well as different kinds of HIF-PHIs, dosing regimens and durations. Hence, more clinical trials are needed to address whether HIF-PHIs and ESAs are differentially effective in iron metabolism.

In addition to evaluating the therapeutic effect, a comprehensive assessment of the adverse event profiles of therapy options is crucial. Several previous meta-analyses did not find significant differences in adverse reactions, especially all-cause mortality and cardiovascular events, between HIF-PHIs and placebo or ESAs (Wang et al., 2020; Zheng et al., 2020). However, two recent phase 3 clinical trials reported that among NDD-CKD patients, the risks of major adverse events, including all-cause mortality, nonfatal myocardial infarction, or stroke, are considerably higher in vadadustat-treated subjects than in darbepoetin alfa-treated subjects (hazard ratio 1.17, 95% CI 1.01–1.36) (Chertow et al., 2021). Recent *in vitro* experiments found that activation of the HIF-1 pathway contributes to vascular calcification, which might exacerbate vessel stiffness and cardiovascular disorders (Mokas et al., 2016; Balogh et al., 2019). In our study, analyses revealed that the profiles of AEs, especially SAEs, in the vadadustat group were similar to those in the placebo or darbepoetin alfa group, except for nausea and diarrhea. Nonetheless, only 4 trials with short-term follow up (ranged from 6–20 weeks) compared vadadustat with placebo, and sample sizes were relatively small. Therefore, the long term effect of vadadustat on cardiovascular system in CKD patients remains uncertain and needs to be further investigated. Although vadadustat treatment was associated with more gastrointestinal events, nausea and diarrhea were not considered to be major safety concerns. Because most events were mild, and the 5 included open-label trials might have a risk of bias against AE reporting. Regarding hyperkalemia, previous meta-analyses have reported that HIF-PHIs could increase the risk of hyperkalemia compared with placebo (Wang et al., 2020). However, the pooled data in our analysis indicated that vadadustat did not increase the rate of hyperkalemia versus placebo. Moreover, vadadustat may even decrease the risk of hyperkalemia compared with darbepoetin alfa. Thus, the uncertain relationship between vadadustat and hyperkalemia needs to be studied further, and serum potassium concentrations need to be closely monitored during treatment.

Our review has several strengths. First, this is the first meta-analysis to evaluate the effects and safety of vadadustat for renal anemia that could provide rigorous evidence for vadadustat as a novel HIF-PHI. Second, only RCTs were included in our study, and all of them were multicenter trials. The inclusion of high-quality studies ensures the quality of our results.

### Limitations

There are several potential limitations that should be noted. First, the numbers and sample sizes of the included studies on vadadustat versus placebo were small, and the efficacy of vadadustat could not be completely assessed. Second, the characteristics of the enrolled studies had inherent clinical heterogeneity. Because of the limited data, a more comprehensive subgroup analysis based on different factors could not be conducted. Third, because of the wide range of vadadustat doses and intervention times, we portray only an overview of the effect and short-term adverse effects of vadadustat. The optimal dosing and treatment strategies for renal anemia are still unknown. Similarly, long-term clinical data on the potential adverse effects of vadadustat on CKD patients are lacking.

## Conclusion

This systematic review and meta-analysis provides evidence that vadadustat could effectively improve the level of Hb and promote iron utilization in CKD patients with anemia. In NDD-CKD patients with anemia, vadadustat is noninferior to darbepoetin alfa. In DD-CKD patients with anemia, the efficacy of vadadustat compared with darbepoetin alfa remains uncertain. Vadadustat is generally well tolerated without increasing the risk of SAEs, especially mortality, cardiac events and nonfatal stroke. However, those findings should be interpreted with caution because of a limited number of studies, heterogeneity and short-term follow-up. More well-designed RCTs with larger sample sizes and long-term follow-up are needed to further investigate the efficacy and safety of vadadustat, especially in DD-CKD populations.

## Data Availability

The original contributions presented in the study are included in the article/Supplementary Material; further inquiries can be directed to the corresponding author.

## Supplementary Material

Supplementary Figure S1 Risk of bias summary: Review authors’ judgments about each risk of bias item for each included study.

Supplementary Figure S2 Major adverse events included all-cause mortality, cardiac events and nonfatal stroke with vadadustat versus placebo or darbepoetin alfa.

Supplementary Figure S3 Risk ratios of nausea for vadadustat versus placebo and darbepoetin alfa.

Supplementary Figure S4 Risk ratios of diarrhea for vadadustat versus placebo and darbepoetin alfa.

Supplementary Figure S5 Risk ratios of hyperkalemia for vadadustat versus placebo and darbepoetin alfa.

Supplementary Figure S6 Change in iron metabolism parameters (hepcidin, ferritin and TIBC) with vadadustat versus placebo or darbepoetin alfa.

Supplementary Figure S7 Risk ratios of the use of rescue medications with vadadustat versus placebo or darbepoetin alfa.

## Abbreviations

AEs, adverse events; CENTRAL, Cochrane Central Register of Controlled Trials; CKD, chronic kidney disease; CNKI, China National Knowledge Infrastructure; DD-CKD, dialysis-dependent CKD; ESAs, erythropoiesis-stimulating agents; EPO, erythropoietin; GRADE, Grading of Recommendation Assessment, Development and Evaluation; Hb, hemoglobin; ∆Hb, change in Hb level from baseline; HIF-PHIs, hypoxia-inducible factor prolyl hydroxylase inhibitors; MD, mean difference; NDD-CKD, nondialysis-dependent CKD; PRISMA, Preferred Reporting Items for Systematic Reviews and Meta-analyses; RBC, red blood cell; RCTs, randomized controlled trials; RRs, risk ratios; SAEs, severe adverse events; SMDs, standardized mean differences; TIBC, total iron-binding capacity.
