# Supplementary material for: Efficacy and Safety of Vadadustat for Anemia in Patients With Chronic Kidney Disease: A Systematic Review and Meta-Analysis
Source: Front Pharmacol. 2022 Jan 18;12:795214. doi: 10.3389/fphar.2021.795214 (PMC8804247; doi:10.3389/fphar.2021.795214)
Supplement: Supplementary file 1 [file DataSheet1.docx]

SUPPLEMENTARY MATERIAL

| **Supplemental Table 1:** Summary of findings | | | | | | | | | |
| --- | --- | --- | --- | --- | --- | --- | --- | --- | --- |
| **The effect and safety of vadadustat treatment for anemia in chronic kidney disease patients** | | | | | | | | | |
| **Patient or population**: Subjects diagnosed with CKD-associated anemia  **Setting**: Randomized controlled trials  **Intervention**: Vadadustat  **Comparison**: Placebo or darbepoetin alfa | | | | | | | | | |
| Outcomes | **Anticipated absolute effects^*^**(95% CI) | | | Relative effect (95% CI) | | № of participants  (studies) | Certainty of the evidence (GRADE) | |  |
|  | **Risk with placebo** | | **Risk with Hb response** |  |  |  |  |  |  |
| Hb response | | 582 per 1,000 | **582 per 1,000** (524 to 652) | **RR 1.00** (0.90 to 1.12) | | 8127 (8 RCTs) | ⨁⨁◯◯ LOW^a,b^ | |  |
| Hb response subgroup - Vadadustat VS. Placebo | 104 per 1,000 | | **548 per 1,000** (279 to 1,000) | **RR 5.27** (2.69 to 10.31) | | 249 (2 RCTs) | ⨁⨁⨁⨁ HIGH^c^ | |  |
| Hb response subgroup - Vadadustat VS. Darbepoetin alfa | 591 per 1,000 | | **568 per 1,000** (532 to 609) | **RR 0.96** (0.90 to 1.03) | | 7878 (6 RCTs) | ⨁⨁◯◯ LOW^a,b^ | |  |
| ∆Hb | | - | MD **0.76 higher** (0.11 higher to 1.40 higher) | - | 948 (6 RCTs) | | ⨁⨁◯◯ LOW^b,d^ | |  |
| ∆Hb subgroup analysis - Vadadustat VS. Placebo | - | | MD **1.28 higher** (0.83 higher to 1.73 higher) | - | | 322 (4 RCTs) | ⨁⨁◯◯ LOW^b,c^ | |  |
| ∆Hb subgroup analysis- Vadadustat VS. Darbepoetin alfa | - | | MD **0.27 lower** (0.52 lower to 0.01 lower) | - | | 626 (2 RCTs) | ⨁⨁⨁◯ MODERATE^b^ | |  |
| AEs | 890 per 1,000 | | **881 per 1,000** (872 to 899) | **RR 0.99** (0.98 to 1.01) | | 8412 (10 RCTs) | ⨁⨁⨁⨁ HIGH | |  |
| SAEs | 556 per 1,000 | | **545 per 1,000** (523 to 567) | **RR 0.98** (0.94 to 1.02) | | 8412 (10 RCTs) | ⨁⨁⨁⨁ HIGH | |  |
| All cause mortality | 146 per 1,000 | | **146 per 1,000** (132 to 162) | **RR 1.00** (0.90 to 1.11) | | 8412 (10 RCTs) | ⨁⨁⨁⨁ HIGH | |  |
| Cardiac events | | 69 per 1,000 | **71 per 1,000** (61 to 83) | **RR 1.03** (0.88 to 1.20) | 8412 (10 RCTs) | | | ⨁⨁⨁⨁ HIGH |  |
| Nonfatal stroke | | 18 per 1,000 | **16 per 1,000** (10 to 28) | **RR 0.92** (0.55 to 1.57) | 7373 (4 RCTs) | | | ⨁⨁⨁◯ MODERATE^b^ |  |
| Hepcidin | | - | MD **36.62 lower** (54.95 lower to 18.3 lower) | - | 4314 (6 RCTs) | | | ⨁⨁⨁◯ MODERATE^b^ |  |
| TIBC | | - | MD **24.38 higher** (13.69 higher to 35.07 higher) | - | 393 (4 RCTs) | | | ⨁⨁◯◯ LOW^b,c^ |  |
| Ferritin | | - | MD **56.24 lower** (77.37 lower to 35.11 lower) | - | 4314 (6 RCTs) | | | ⨁⨁⨁◯ MODERATE^b^ |  |
| Nausea | | 71 per 1,000 | **86 per 1,000** (74 to 100) | **RR 1.21** (1.04 to 1.40) | 8017 (8 RCTs) | | | ⨁⨁⨁⨁ HIGH |  |
| Diarrhea | | 98 per 1,000 | **133 per 1,000** (117 to 150) | **RR 1.35** (1.19 to 1.53) | 7966 (7 RCTs) | | | ⨁⨁⨁⨁ HIGH |  |
| Hyperkalemia | | 105 per 1,000 | **88 per 1,000** (78 to 101) | **RR 0.84** (0.74 to 0.96) | 8261 (8 RCTs) | | | ⨁⨁⨁⨁ HIGH |  |
| ESA rescue | | 131 per 1,000 | **74 per 1,000** (39 to 137) | **RR 0.56** (0.30 to 1.04) | 6850 (7 RCTs) | | | ⨁⨁⨁◯ MODERATE^b^ |  |
| RBC transfusion | | 17 per 1,000 | **17 per 1,000** (11 to 27) | **RR 0.99** (0.62 to 1.58) | 6850 (7 RCTs) | | | ⨁⨁⨁⨁ HIGH |  |
| ***The risk in the intervention group** (and its 95% confidence interval) is based on the assumed risk in the comparison group and the **relative effect** of the intervention (and its 95% CI). **CI:** Confidence interval; **RR:** Risk ratio; **MD:** Mean difference | | | | | | | | | |
| **GRADE Working Group grades of evidence** **High certainty:** We are very confident that the true effect lies close to that of the estimate of the effect. **Moderate certainty:** We are moderately confident in the effect estimate: the true effect is likely to be close to the estimate of the effect, but there is a possibility that it is substantially different. **Low certainty:** Our confidence in the effect estimate is limited: the true effect may be substantially different from the estimate of the effect. **Very low certainty:** We have very little confidence in the effect estimate: the true effect is likely to be substantially different from the estimate of effect. | | | | | | | | | |

**Explanations**

a. Enrolled RCT was at high risk of bias for incomplete data and thereforerated down for risk of bias.

b. The pooled result with substantial heterogeneity

c. The total number of patients included in a systematic review was less than the number of patients generated by a conventional sample size calculation for a single adequately powered trial; therefore, the RCT was rated down for imprecision.

d. For ∆Hb, Egger’s test (*P=* 0.02) and Begg’s test (*P=* 0.26) suggested that there might be publication bias.


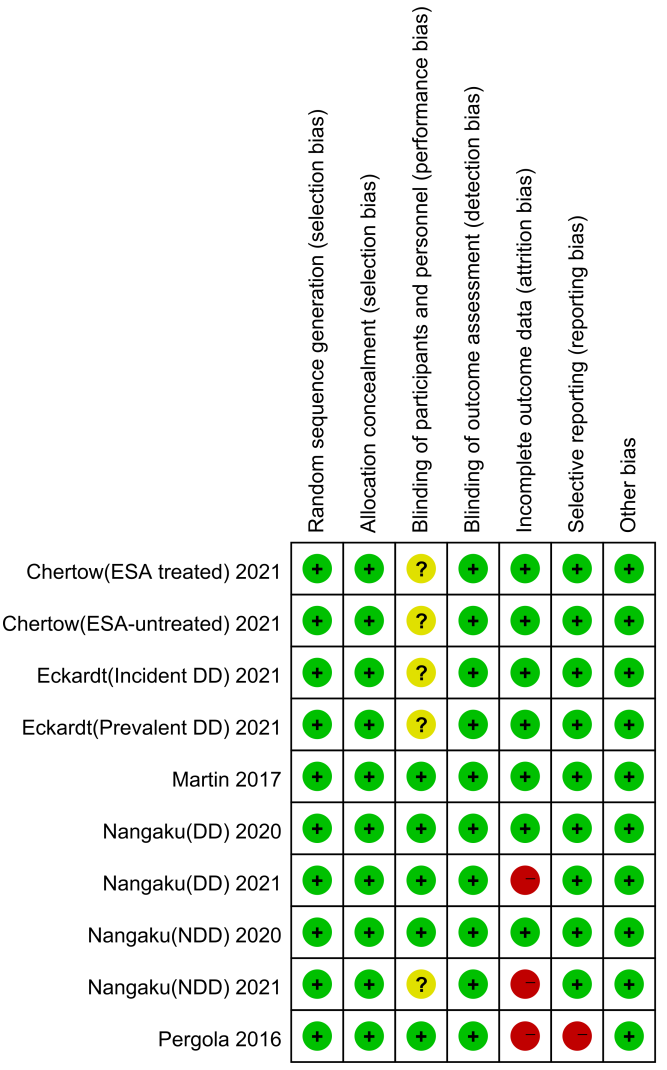


**Supplementary Figure S1.**Risk of bias summary: review authors’ judgments about each risk of bias item for each included study.


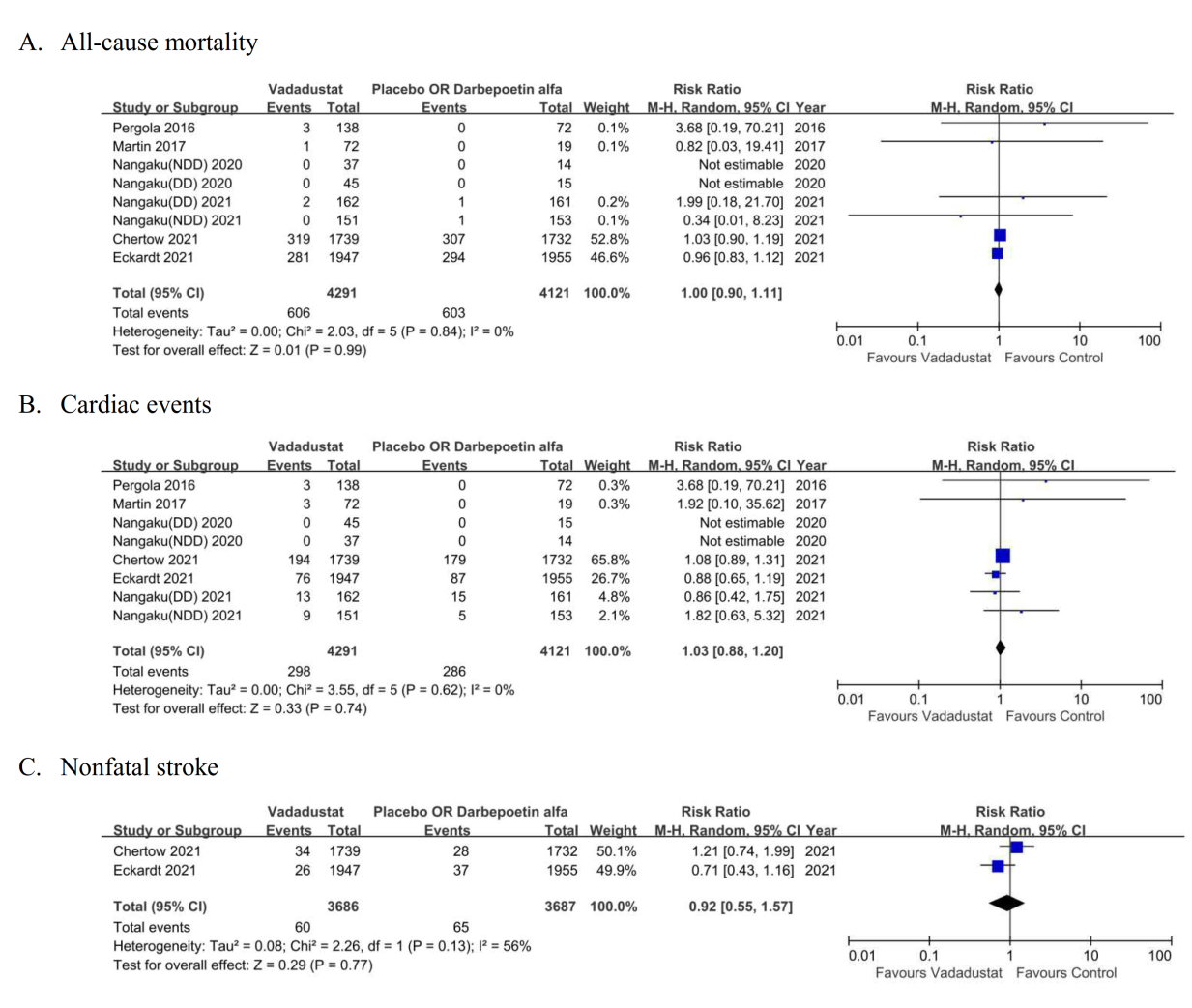


**Supplementary Figure S2.** Major adverse events included all-cause mortality, cardiac events and nonfatal stroke with vadadustat versus placebo or darbepoetin alfa.


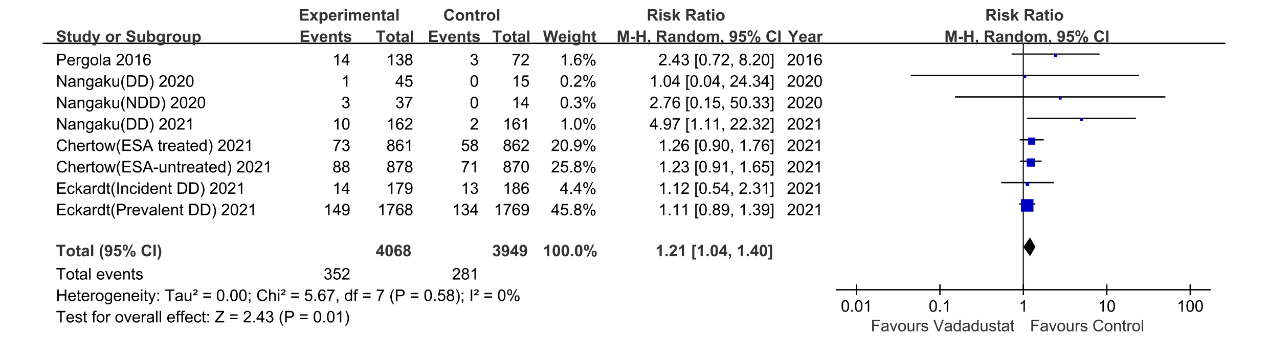


**Supplementary Figure S3.**Risk ratios of nausea for vadadustat versus placebo and darbepoetin alfa.


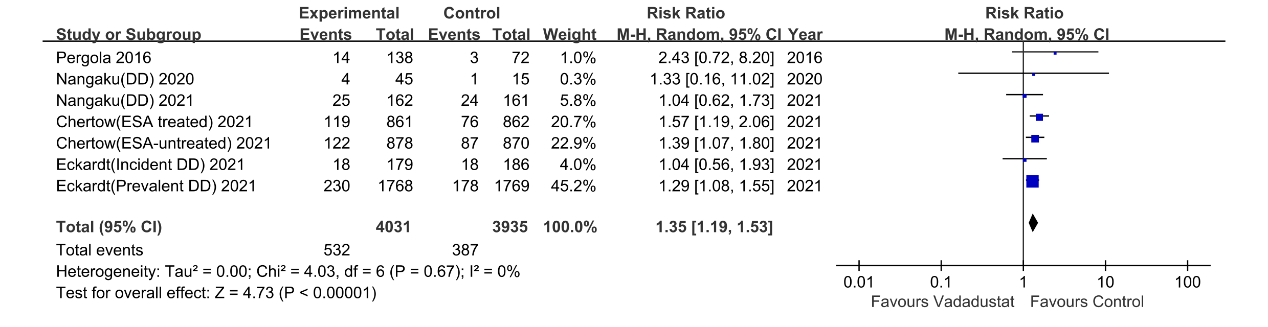


**Supplementary Figure S4.**Risk ratios of diarrhea for vadadustat versus placebo and darbepoetin alfa.


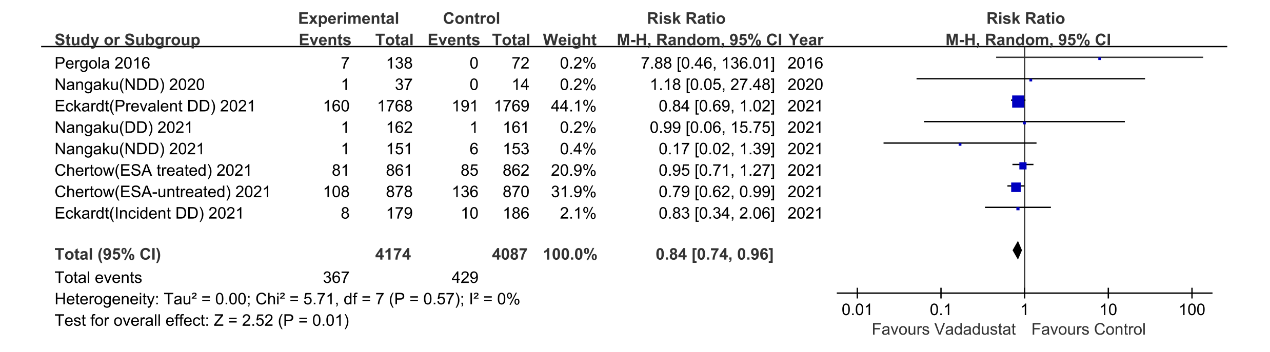


**Supplementary Figure S5.**Risk ratios of hyperkalemia for vadadustat versus placebo and darbepoetin alfa.


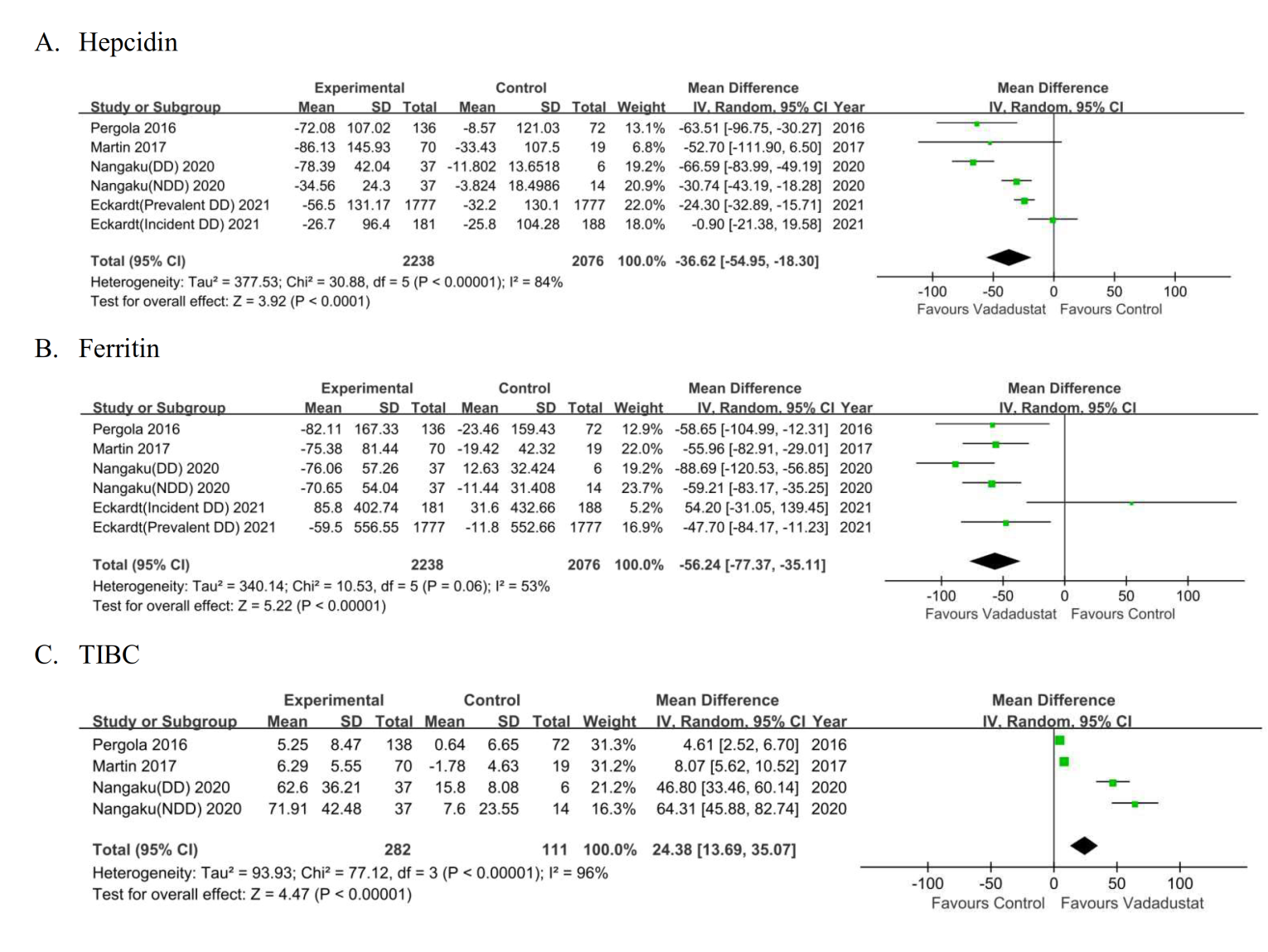


**Supplementary Figure S6.**The change in iron metabolism parameters (hepcidin, ferritin and TIBC) with vadadustat versus placebo or darbepoetin alfa.


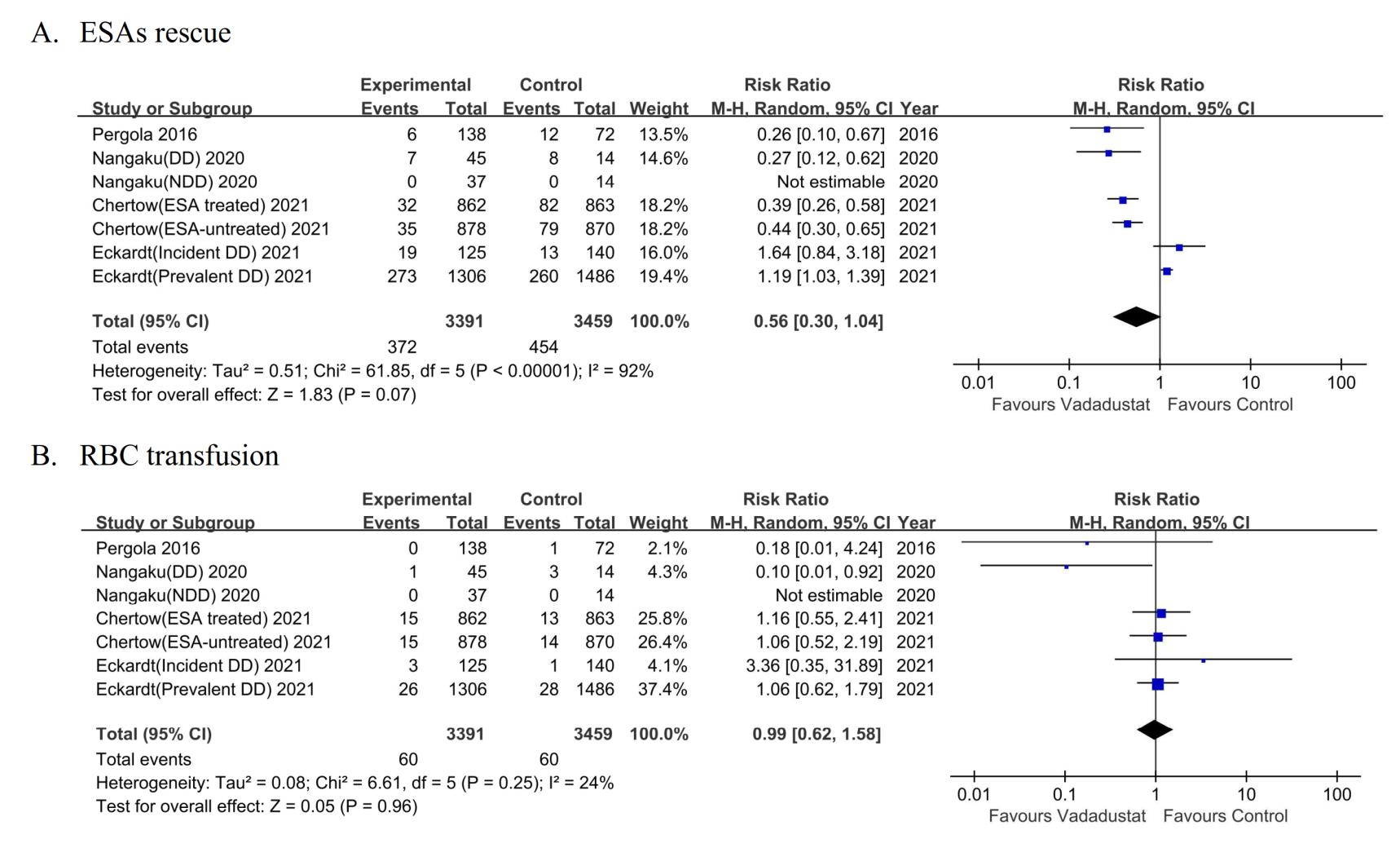


**Supplementary Figure S7.**Risk ratios of the use of rescue medications with vadadustat versus placebo or darbepoetin alfa.
